# Phase Evolution, Filler-Matrix Interactions, and Piezoelectric Properties in Lead Zirconate Titanate (PZT)-Filled Polymer-Derived Ceramics (PDCs)

**DOI:** 10.3390/ma13071520

**Published:** 2020-03-26

**Authors:** Franziska Eichhorn, Simone Kellermann, Ulf Betke, Tobias Fey

**Affiliations:** 1 Institute of Glass and Ceramics, Department of Materials Science and Engineering, University of Erlangen-Nürnberg, Martensstraße 5, 91058 Erlangen, Germany; franziska.eichhorn@fau.de (F.E.); kellermann_simone@web.de (S.K.); 2 Institute for Materials and Joining Technology—Nonmetallic Inorganic Materials and Composites, Otto-von-Guericke-University Magdeburg, Große Steinernetischstraße 6, 39104 Magdeburg, Germany; ulf.betke@ovgu.de; 3 Frontier Research Institute for Materials Science, Nagoya Institute of Technology, Nagoya 466-8555, Japan

**Keywords:** piezoelectric material, polymer-derived ceramic (PDC), lead zirconate titanate (PZT), composite material, filler matrix interaction

## Abstract

PZT-silsesquioxane-based 0-3 hybrid materials are prepared by mixing lead zirconate titanate (Pb(Zr,Ti)O_3_; PZT) powder with a [R-SiO_3/2_]_n_ (R = H, CH_3_, CH=CH_2_, C_6_H_5_) silsequioxane preceramic polymer. A PZT load up to 55 vol.% can be reached in the final composite. The piezoelectric and mechanical properties are investigated as a function of the filler content and are compared with theoretical models and reference samples made of the pure preceramic polymer or PZT filler. The piezoelectric response of the composites, as expressed by the relative permittivity and the piezoelectric coefficients d_33_ and g_33_, increases with an increasing PZT content. The bending strength of the composites ranges between 15 MPa and 31 MPa without a clear correlation to the filler content. The thermal conductivity increases significantly from 0.14 W∙m^−1^∙K^−1^ for the pure polymer-derived ceramic (PDC) matrix to 0.30 W∙m^−1^∙K^−1^ for a sample containing 55 vol.% PZT filler. From X-ray diffraction experiments (XRD), specific interactions between the filler and matrix are observed; the crystallization of the PDC matrix in the presence of the PZT filler is inhibited; conversely, the PDC matrix results in a pronounced decomposition of the filler compared to the pure PZT material.

## 1. Introduction

Transducers such as sensors and actuators based on piezoelectric ceramic-polymer composites (smart materials) offer a high potential for high tech systems [1]. These composite materials provide superior mechanical properties, such as elasticity, compared to conventional ceramic materials and can be optimized with respect to the piezoelectric properties as a function of the filler content. Due its high piezoelectric response and excellent electromechanical properties at the morphotropic phase boundary (MPB), lead zirconate titanate Pb(Zr*_x_*Ti_1−*x*_)O_3_ (PZT) is mostly used as filler in these types of composite materials. Pb(Zr*_x_*Ti_1−*x*_)O_3_ exists in three crystallographic polymorphs—tetragonal, rhombohedral, and cubic PZT [2]—whereas the phase stability depends on the temperature and Zr:Ti ratio, respectively. At the MPB the tetragonal and rhombohedral Pb(Zr_1−x_Ti_x_)O_3_ phases coexist [3] and the number of the polarization directions increases to 14 allowing an electrical-mechanical energy conversion efficiency up to 50% [2,4]. At room temperature the MPB in PZT is found for a Zr:Ti molar ratio of 53:47. 

The properties of a composite material are determined by the number and the characteristics of the individual components as well as their composition and the connectivity between them. Newnham et al. introduced the concept of 0-3 connectivity, which describes isolated ceramic particles (no connectivity) filled into a three-dimensional polymer matrix (connectivity in all three spatial directions) [5]. Ceramic-polymer 0-3 hybrid materials feature several advantages in comparison to other composite materials: a simple and effective manufacturing process, tailored properties by varying the volume fraction of the ceramic filler and flexible control of shape by established polymer forming techniques. 

A number of studies were published on 0-3 composites consisting of different polymers and piezoelectric ceramics [1,6,7,8,9,10]. All these composites are more flexible in comparison to the pure (piezoelectric) ceramic material. Piezocomposites are binary systems composed of piezoceramic filler particles with a dielectric constant ε_2_ dispersed in a continuous polymer matrix with a dielectric constant ε_1_, whereas ε_2_ > ε_1_ [11]. With respect to the mechanical properties, this takes advantage of the flexibility and lower density of a polymer as a matrix, which is favorable for mechanical damping. However, polymers have a low dielectric constant, and, consequently, the piezoelectric response of the piezocomposite is a function of the amount of ceramic filler material Φ [11].

The combination of a small fraction of piezoelectric ceramic particles in a polymer matrix results in soft composites with limited piezoelectric properties. Moreover, the inter-connectivity of the piezoelectric particles is poor, and, hence, such composites show only a weak piezo electric effect. Heat generation at the PZT polymer interface, due to the friction between the filler and matrix, increases the passive damping at the cost of the mechanical energy. In addition, only a few polymer materials, such as polyvinylidenedifluoride (PVDF), possess a sufficient dielectric constant and dielectric strength to permeate the electric field necessary for the poling of the piezoelectric filler particles without dielectric breakdown and facilitating the active vibration damping composites [7,12].

In 0-3 ceramic-polymer composites, the (spherical) dielectric filler particles are dispersed in a continuous matrix material. The dielectric particles can be polarized by an external electrical field, whereas an individual particle can be represented by a single dipole moment. This dipole moment locally modifies the applied electrical field in the surrounding matrix medium. For small volume fractions of dielectric filler particles, equivalent to a long particle-particle distance, the mutual influence of the dipole moment of neighboring particles is negligible. Therefore, the prediction of field ratios by using the isolated sphere theory is physically reasonable. For increasing the amount of dielectric filler, it is no longer valid to ignore the cooperative effects between the electrical fields of neighboring particles [11]. Different models exist for the prediction of the piezoelectric properties of 0-3 composite materials, which usually include the permittivity of the individual matrix and filler phase as well as the volume fraction between filler and matrix, but ideally assume spherical particles [1]. A widely applied model from Yamada includes an anisotropy in particle shape and a distinct alignment of the piezoelectric filler particles along the electric field [10]. A model formulated by Jayasundere includes an interaction between neighbouring filler particles but on a basis of the assumption of the ideal spherical shape [11]. However, both models, Yamada and Jayasundere, are well suited to predict the piezoelectric properties of polymer-PZT composites [1,13].

In this study, the processing and characterization of new 0-3 piezoelectric composites based on a preceramic silsesquioxane polymer is reported. Preceramic polymers can be shaped at a moderate temperature and subsequently crosslinked at temperatures between 100 and 150 °C into the respective thermoset. Further increasing the temperature to between 600 and 800 °C under inert atmosphere results in the loss of the organic moieties in the polymer backbone (pyrolysis) and the formation of an amorphous ceramic. At temperatures > 1000 °C, the amorphous ceramic starts to crystallize. Siloxane-based preceramic polymers result in the formation of SiOC ceramics initially, which finally crystallize into SiO_2_ and SiC [14].

Within this work, the volume fraction of the PZT filler is varied between 0 vol.% and 55 vol.%, and, in addition, the influence of a pyrolysis temperature between 750 and 1250 °C is investigated. The experimental results for the piezoelectric parameters relative permittivity, and piezoelectric charge constant d_33_ were compared with the results from the theoretical models of Yamada, Jayasundere, and Lichtenecker [1,2,10,11,13]. Mechanical properties such as bending strength as well as the thermal conductivity were measured and analyzed in dependence of the filler content of the sample and heat treatment processing.

## 2. Materials and Methods

### 2.1. Sample Preparation

The PZT-powder (NCE51, Noliac Ceramics s.r.o., Livrice, Czech Republic; particle size: 1.8 µm, see Figure S3, Supporting Information) was heat treated at 700 °C for 2 h in air for debinding. Afterwards, 32 vol.% of debinded PZT powder was dispersed in a solution of 0.2 vol.% stearic acid (Riedel de Haen, Seelze, Germany) in 67.8 vol.% *n*-hexane (Merck KGaA, Darmstadt, Germany). The resulting mixture was roller-milled for 24 h. Afterwards, the hydrophobized PZT powder was obtained by the evaporation of the solvent and final drying at 160 °C for 6 h. To produce the 0-3 PZT-silsesquioxane hybrid materials, the hydrophobized PZT powder was mixed with the preceramic polymer SILRES H62C (Wacker Chemie AG, Burgdorf, Germany) using the vacuum stirrer Koala (Mestra, Passau, Germany). The mass obtained after vacuum mixing was degassed and homogenized in the vacuum mixer for 30 min. H62C is a silsesquioxane oligomer with the general composition [R-SiO_3/2_]_n_ (R = H, CH_3_, CH=CH_2_, C_6_H_5_) and contains ≡Si-H as well as ≡Si-CH=CH_2_ moieties. The crosslinking can be performed at moderate temperature by a hydrosilylation reaction; the necessary platinum catalyst is included in the H62C material.

The amount of PZT in the mass was varied between 0 and 55 vol.%, whereas mixtures with a filler content larger than 50 vol.% were kneaded in a roller mill (EXAKT Advanced Technologies GmbH, Norderstedt, Germany). Finally, the homogenized and degassed PZT-H62C mixture was filled into self-made silicon molds (Elastosil M 4643 A+B, Wacker Chemie AG, Burgdorf, Germany). The curing of the PZT-H62C mixture was performed at 140 °C for 3 to 6 h. After demolding, the samples were heated to 200 °C for 5 h. The selected samples were finally pyrolyzed in Ar atmosphere at 750 or 1250 °C, respectively, for 2 h. To avoid the evaporation of PbO from the PZT filler during the heat treatment, the samples were heated together with a mixture of PbO, ZrO_2_ and PbZrO_3_ in a closed crucible. 

The reference samples made of the pure PZT ceramic were prepared by injection molding from a mass containing 48.1 vol.% PZT, 47.2 vol.% carnauba wax (Carl Roth GmbH, Karlsruhe, Germany), and 4.7 vol.% Granopent paraffin (Carl Roth GmbH, Karlsruhe, Germany). The samples without PZT filler were prepared by crosslinking and pyrolyzing pure H62C at the conditions stated previously.

### 2.2. Metallization and Polarization of the Samples

For the characterization of the piezoelectric response, the samples were cut into two different geometries: rod-like specimens with a length:width:height ratio of 13:3:1 and cylindrical samples with a diameter:height ratio of 10:1. The top and bottom faces were polished using a 3-µm diamond dispersion and were subsequently metallized by coating with conductive silver (Acheson Silber DAG 1415, Plano GmbH, Wetzlar, Germany). After drying, the metallized samples were put in between two copper electrodes and placed into a silicone oil bath (Wacker Chemie AG, Burgdorf, Germany). The polarization was performed at a temperature of 130 °C and the electric field was increased within 30 min to a final strength of 2 kV∙mm^−1^. Afterwards, the samples were cooled to room temperature within three hours while keeping the electric field constant. The optimal polarization conditions required for the PZT-polymer composite materials within this work were estimated by the Kura Kawa theory [7,15].

### 2.3. Sample Characterization

The analysis of the sample microstructure was performed by scanning electron microscopy with an acceleration voltage of 25 kV (SEM, Quanta 2000, FEI, Hillsboro, OR, USA) after grinding the specimen surface with 180-, 600-, 2000-grit SiC paper and polishing with a 3-µm diamond dispersion. The quantitative phase composition in the composite materials was analyzed by powder X-ray diffraction, XRD (D500 diffractometer, Siemens AG, Karlsruhe, Germany) using copper Kα_1_/α_2_ radiation after ball-milling the respective sample for 5 min at 300 rpm. The grinded powder was filled into a backloading sample holder and measured in a *θ*/*θ* reflection geometry with a 2*θ* range from 5° to 70°. The obtained diffraction patterns were analyzed by the Rietveld technique using the Topas Academic 5 package (Coelho Software, Brisbane, Australia).

The thermogravimetric analysis of selected PZT-polysilsesquioxane composites was carried out up to 1300 °C with a heating rate of 5 K/min under flowing Nitrogen using a STA429 device (Netzsch GmbH, Selb, Germany). Prior to the sample investigation, a correction thermogravimetry (TG) curve was recorded with the respective empty alumina crucible, which was subtracted from the final measurement. 

The pycnometric sample density was determined by He pycnometry (AccuPyc II 1340, Micromeritics, Norcross, GA, USA) and compared to the geometric density of the samples. The total porosity of the specimens was calculated from the ratio of geometric density and pycnometric density.

The bending strength was determined on bars with 2 × 2.5 × 25 mm^3^ by the four-point bending test using an Instron Model 5565 testing machine (Instron GmbH, Pfungstadt, Germany). For each sample series, 15 specimens were measured and the bending strength *σ_f_* was calculated from the fracture force *F*, the sample width *b* and height *h*, and the distance between the four rolls of the four-point bending setup (*l* = top, *L* = bottom) according to Equation (1):

(1) $$\sigma_{f}=\frac{3\times F\times \left(L-l\right)}{2\times b\times h}$$

The average bending strength was calculated by the Maximum Likelihood method.

The thermal conductivity of the composite materials was determined on cylindrical samples by the hot-plate method at a temperature difference of 20 K (40 °C hot plate temperature against room temperature). For each sample series, five specimens were measured and averaged. The collected data were compared to theoretical values estimated by the Landauer model based on the effective percolation theory [16,17].

For the characterization of the piezoelectric properties of the samples, the relative permittivity as well as the charge constant d_33_ were determined using the Berlincount method with a piezometer PM 300 at a fixed frequency of 110 Hz (Piezo Test, London, UK). The sample was mounted between the oscillating contact pads of the measurement device with a contact force of 2 N. The experimental results were compared with theoretical permittivity values calculated by the Yamada model for anisotropic particles [10], the model of Jayasundere [11], and the model of Lichtenecker [1].

## 3. Results and Discussion

### 3.1. Microstructure Evolution in PZT-Polymer and PZT-PDC Composites

**Figure 1:**
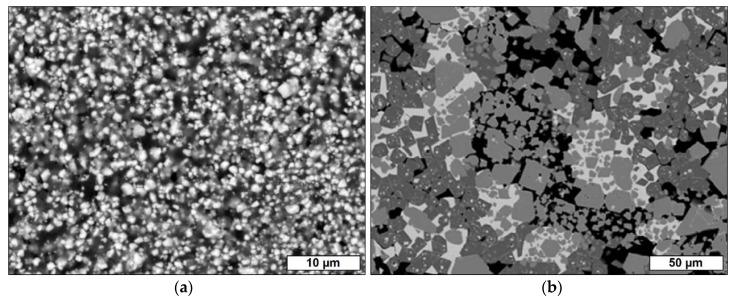
Scanning electron microscopy (SEM) micrographs of selected Pb(Zr,Ti)O_3_ (PZT)-polymer composites with a filler content of 40 vol.% PZT (**a**) after the crosslinking of the silsesquioxane resin at 200 °C and (**b**) after heat treatment processing at 1250 °C.

From SEM micrographs, the formation of a percolating network of PZT filler particles in the samples cured at 200 °C is observed for PZT loads exceeding 50 vol.% (Figure S1, Supporting Information). For lower volume fractions of PZT, the individual particles are separated through the polymer matrix material without interconnection between the filler species (Figure 1a). The mean particle diameter of the PZT filler in the composite samples crosslinked at 200 °C is 1.8 µm.

**Figure 2:**
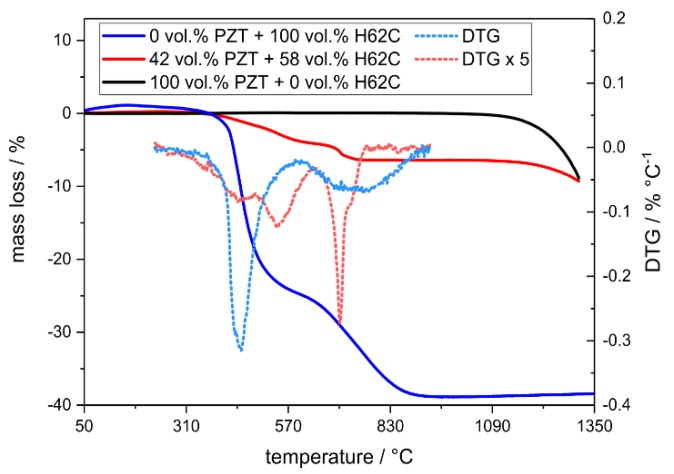
Thermogravimetry of the pure, hydrophobized PZT powder (black line), a composite material containing 42 vol.% PZT (red line) and of the pure H62C silsesqioxane polymer (blue line). The dotted lines show the first derivative of the respective thermogravimetry data (DTG).

During the heat treatment of the composites at elevated temperatures between 500 and 1250 °C, microstructural changes are observed (Figure S2, Supporting Information). The ceramization of the pure H62C preceramic polymer occurs in two subsequent steps in the temperature range between 400 and 900 °C according to thermogravimetry (TG) analyses (Figure 2), whereas the pure H62C preceramic polymer gives a ceramic yield of approximately 62 wt.%. The presence of the PZT filler particles changes the decomposition behavior of the preceramic polymer; the ceramization now takes place in four consecutive reactions in the temperature range between 400 and 750 °C. Consequently, the PZT filler alters the decomposition reactions in the PDC matrix, resulting in a complete ceramization at a temperature 150 °C lower than for the pure H62C material. After thermal treatment at 500 and 750 °C, the microstructure of the samples is similar to the structure of the initial composites crosslinked at 200 °C. Nevertheless, the matrix phase is made up of a mixture of polymer and already formed amorphous PDC material. With respect to the size and shape of the PZT filler particles, no significant changes were observed.

The pure PZT filler is stable up to approximately 1000 °C with respect to the TG data; no significant weight change is observed. However, in the temperature regime between 1000 and 1300 °C, a weight loss of 9 wt.% is observed as a consequence of the decomposition of the PZT powder due to the evaporative loss of PbO [18]. 

From the SEM micrographs, a significant grain growth of the filler particles is observed for a heat treatment at 1000 °C and especially at 1250 °C (Figure S2; Supporting Information). For a pyrolysis at 1250 °C, at least three separate phases are formed with respect to the grey levels within the SEM image (Figure 1b). This is in agreement with the TG results, which indicate a decomposition of the PZT phase above 1000 °C.

**Figure 3:**
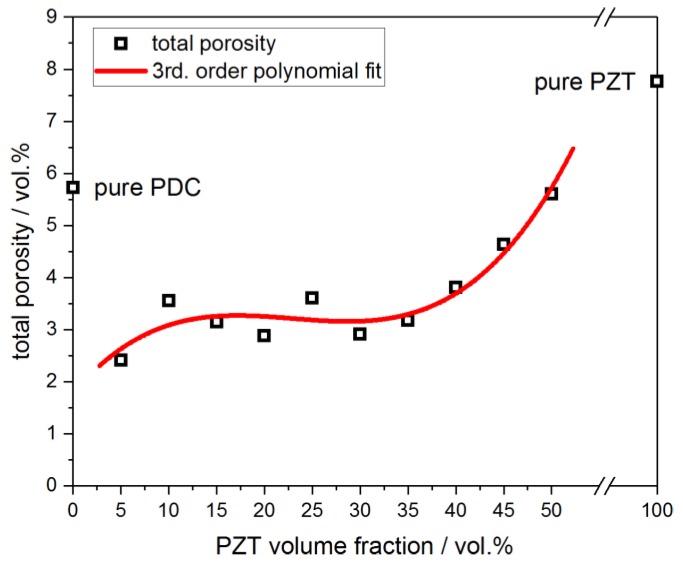
Porosity of the PZT-polymer composites crosslinked at 200 °C as a function of the PZT filler volume fraction.

The porosity, as determined by the ratio of the geometric sample density and the pycnometric bulk density of the material, ranges between 2.4 vol.% and 5.6 vol.% for the PZT-silsesquioxane composites crosslinked at 200 °C (Figure 3). For filler loads between 5 and 35 vol.%, no significant variation in the porosity is detected with an average of 3.1 vol.%. However, in samples with a PZT load above 35 vol.%, the porosity increases significantly up to 5.6 vol.% for the specimen containing 50 vol.% PZT filler. This increase in porosity is correlated to the increasing difficulty in homogenization and especially the degassing of the initial PZT-H62C mixture for high filler loads.

### 3.2. Phase Evolution in PZT-Polymer and PZT-PDC Composites

**Figure 4:**
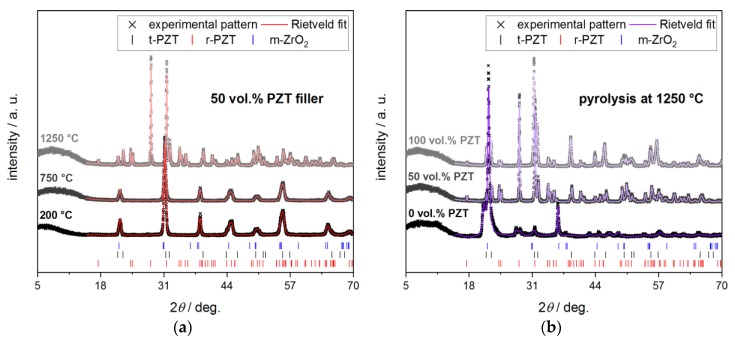
Powder XRD patterns for PZT-polymer and PZT-PDC composites together with the corresponding Rietveld fits. (**a**) The variation in the heat treatment temperature at a constant PZT load of 50 vol.%; (**b**) the variation in the PZT volume fraction for a pyrolysis temperature of 1250 °C.

**Figure 5:**
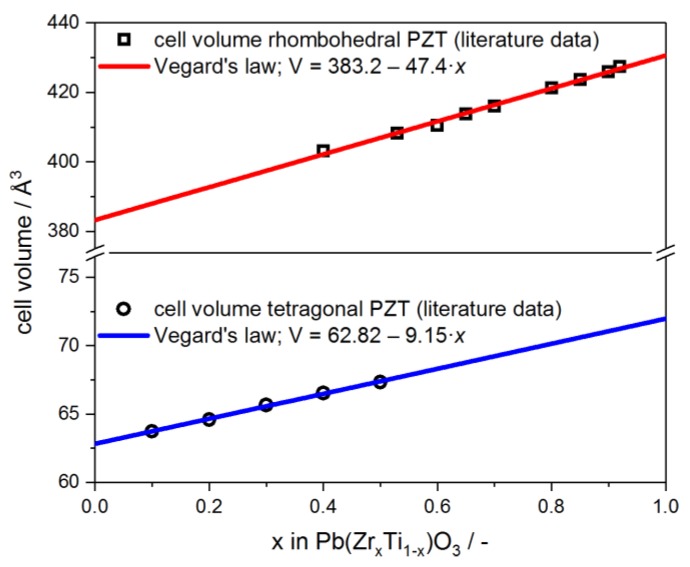
Literature data of the unit cell volume as a function of the Zr content *x* in rhombohedral (□) and tetragonal Pb(Zr*_x_*Ti_1−*x*_)O_3_ (○). The data were fitted by Vegard’s law, which was subsequently applied in determining the Zr content *x* in the PZT filler from its unit cell volume.

The phase composition of the composite samples has been determined as a function of the filler load and the pyrolysis temperature by powder XRD and Rietveld analysis. The results are summarized in Table 1. The diffractograms of selected samples are shown in Figure 4, together with the corresponding Rietveld fits. The Zr concentration *x* in the respective Pb(Zr*_x_*Ti_1−*x*_)O_3_ phases has been determined from their unit cell volume by applying Vegard’s law [19] on structural data for rhombohedral and tetragonal PZT from the literature [20,21,22,23,24]. From this linear relationship, the Zr content *x* in the PZT filler of the respective sample could be estimated (Figure 5).

The pure PZT filler consists of a mixture of rhombohedral Pb(Zr*_x_*Ti_1−*x*_)O_3_ (39 wt.%) and tetragonal Pb(Zr*_x_*Ti_1−*x*_)O_3_ (61 wt.%), and, for both phases, a Zr content of *x* = 0.50 has been determined after a heat treatment at 200 °C. This is in good accord with a (commercial) PZT material close to the MPB with an optimized electrical-mechanical energy conversion efficiency [3,4]. The pure polymer matrix crosslinked at 200 °C is completely amorphous. The crystallization of the PDC is observed for a specimen without PZT filler heat treated at 1250 °C; in this case, SiO_2_, as the cristobalite polymorph, is only detected in the crystalline phase.

For the composite samples containing 50 vol.% of PZT filler, which were heat treated at 200 and 750 °C, respectively, no significant changes in the phase composition are detected. The PZT filler remains stable under these conditions and is still comprised of a mixture of rhombohedral and tetragonal Pb(Zr*_x_*Ti_1−*x*_)O_3_ with a Zr content *x* in a tight range between 0.515 and 0.534 for both phases. Consequently, neither filler decomposition nor the detrimental interactions between the PZT filler and the PDC matrix occur.

This behavior changes significantly for samples heat treated at a temperature of 1250 °C. For the pure PZT filler, a decomposition reaction accompanied by the formation of 39 wt.% monoclinic ZrO_2_ is observed. Only traces of 1 wt.% are detected for the rhombohedral polymorph of Pb(Zr*_x_*Ti_1−*x*_)O_3_ and the phase content of the tetragonal Pb(Zr*_x_*Ti_1−*x*_)O_3_ amounts to 60 wt.%. However, the unit cell volume of 64.9 Å^3^ for the tetragonal Pb(Zr*_x_*Ti_1−*x*_)O_3_ phase is significantly smaller compared to the initial PZT filler material (67.4 Å^3^). This indicates a considerable depletion of Zr in the Pb(Zr*_x_*Ti_1−*x*_)O_3_ phase, which could be quantified to a Zr content *x* of 0.23 according to Vegard’s law. This is in good agreement to the depletion of the rhombohedral polymorph of Pb(Zr*_x_*Ti_1−*x*_)O_3_, which is the stable phase only for higher Zr contents *x*. The driving force of the Pb(Zr*_x_*Ti_1−*x*_)O_3_ decomposition is the volatization of lead(II)-oxide, PbO, which is known to occur for PZT materials at an elevated temperature [18]. Consequently, the decomposition reaction of the PZT material can be formulated according to Equation (2) by introducing a conversion factor *z*, which is the amount of released PbO. In addition, it is assumed that the decomposition reaction yields ZrO_2_ rather than TiO_2_, as no separate, binary titanium-oxygen phase is observed in the Rietveld analyses:

PbZr*_x_*Ti_1*−x*_O_3_ → (1−*z*) PbZr_(*x−z*)/(1*−z*)_Ti_(1*−x*)/(1*−z*)_O_3_ + *z* PbO ↑ + *z* ZrO_2_ (2)

For the PZT material treated at 1250 °C with an initial *x* = 0.5, a conversion factor *z* = 0.35 is in good agreement with the Zr content of 0.23, as calculated from the XRD data for the Pb(Zr*_x_*Ti_1−*x*_)O_3_ phase. However, if the expected weight fractions of Pb(Zr_0.23_Ti_0.77_)O_3_ and ZrO_2_ are calculated under consideration of Equation (2), the amount of ZrO_2_ is clearly underestimated. This can be justified by the formation of significant amounts of amorphous material, which could be either non-crystalline Pb(Zr_0.23_Ti_0.77_)O_3_ or amorphous TiO_2_ formed by further PbO loss from the Pb(Zr_0.23_Ti_0.77_)O_3_ phase. The phase composition determined by Rietveld analysis (39 wt.% ZrO_2_ and 61 wt.% Pb(Zr*_x_*Ti_1−*x*_)O_3_) can be explained by assuming a decomposition according to Equation (2) and the formation of a Pb(Zr_0.23_Ti_0.77_)O_3_ phase, which consists of 67% of non-crystalline material.

For the PZT-PDC composite material with an initial filler load of 50 vol.% and heat treated at 1250 °C, the phase evolution differs significantly from the behavior of both the pure PZT filler and the pure PDC matrix, respectively (Table 1). In comparison to the pure matrix material, no crystallization of the amorphous PDC into cristobalite or another crystalline Si-containing phase is observed. Consequently, the PZT filler is effective at inhibiting the crystallization of the PDC matrix. Compared to the phase evolution of the pure PZT material, the Pb(Zr*_x_*Ti_1−*x*_)O_3_ filler in the PZT-PDC composite is decomposed to a significantly larger extent due to the volatization of PbO. This is manifested in both a higher amount of formed ZrO_2_ with 81 wt.% and a smaller weight fraction of residual tetragonal Pb(Zr*_x_*Ti_1−*x*_)O_3_ of 19 wt.% (Table 1). In addition, the Pb(Zr*_x_*Ti_1−*x*_)O_3_ phase is significantly more depleted in Zr with a Zr content *x* of 0.095. With respect to Equation (2), this is equivalent to a conversion factor *z* of 0.45. Again, the phase composition estimated by Equation (2) differs from the actual Rietveld results, which could be explained by the formation of significant amounts of non-crystalline material.

From the XRD investigations, no direct proof of the formation of (crystalline) ternary oxide phases containing ZrO_2_ or TiO_2_ from the PZT filler and SiO_2_ from the PDC matrix is found. Nevertheless, a distinct interaction between the decomposing PZT filler and the PDC matrix at an elevated temperature can be deduced from the finding of a retarded crystallization of SiO_2_ in the PDC matrix, on the one hand side, and an increased rate of decomposition for the PZT filler, on the other. It is likely that the formation of silica-titania composites is the driving force of both, the increased decomposition of PZT, and the inhibited crystallization of SiO_2_ from the PDC matrix. However, additional experiments, especially for a structural elucidation of the amorphous parts of the PZT-PDC composites, such as solid-state nuclear magnetic resonance (NMR) measurements, and/or vibrational spectroscopy, are necessary.

### 3.3. Mechanical and Thermal Properties of PZT-Polymer Composites

The bending strength of the pure H62C polymer after curing at 200 °C is 31 MPa, which is in good agreement to data from the manufacturer (30 MPa after curing at 150 °C for 16 h; Wacker Chemie AG). The bending strength of the pure PZT filler sintered at 1250 °C is 43 MPa. This is significantly lower than the values from the literature ranging between 60 MPa and 86 MPa for polycrystalline PZT ceramics [25,26]. The reason for this is most likely the partial decomposition of the PZT material during the sintering conditions resulting in the formation of porosity, on the one hand, and of significant amounts of zirconia, on the other. Large amounts of ZrO_2_ within the microstructure of PZT ceramics exceeding 10 vol.% are known to reduce their strength by approximately 50 % due to the formation of microcracks [27].

**Figure 6:**
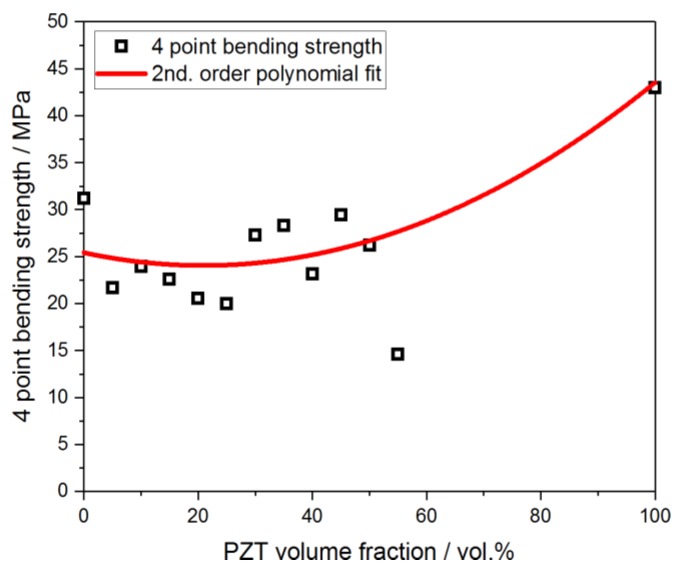
Four-point bending strength of PZT-polymer composites cured at 200 °C as a function of the filler content.

The bending strength of the PZT-polymer composites after curing at 200 °C ranges between 20 MPa and 29 MPa, whereas no direct correlation with the PZT load is observed (Figure 6). The strength of the composite material with the highest PZT content of 55 vol.% should be considered as an outlier as the homogenization of the PZT-H62C mass was considerably hindered due to the high solid content of the mixture. This finding is in good accord with the porosity of the sample with 55 vol.% PZT, which is the highest value in the sample series with 5.6 vol.%.

**Figure 7:**
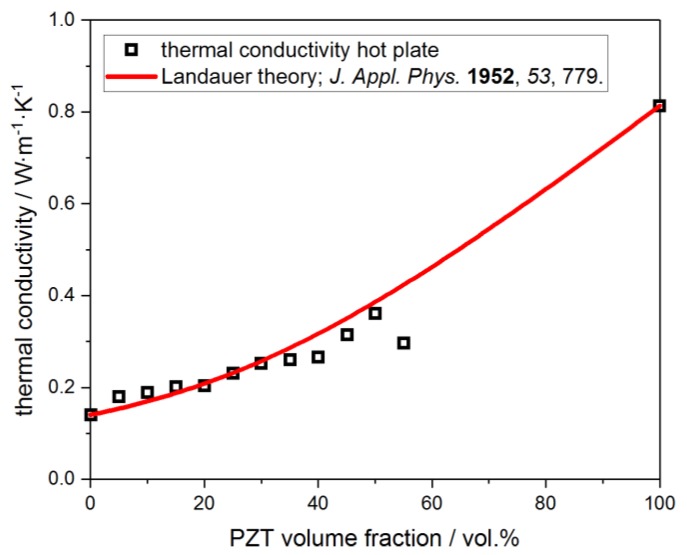
Experimental thermal conductivity (hot plate technique) of PZT-polymer composites cured at 200 °C as a function of the filler content. The observed data (□) fits well to the values extrapolated by the Landauer theory (red line).

The thermal conductivity λ of the pure H62C polymer after curing at 200 °C is 0.14 W∙m^−1^∙K^−1^, which is in good agreement with data from the manufacturer (0.2 W∙m^−1^∙K^−1^ after curing at 150 °C for 16 h; Wacker Chemie AG). For the pure PZT material, after sintering at 1250 °C, a λ of 0.81 W∙m^−1^∙K^−1^ is found, which is lower compared to the literature data of 1.2 W∙m^−1^∙K^−1^ for the room temperature thermal conductivity of pure PZT [28]. Consequently, the thermal conductivity of the PZT-polymer composites after curing at 200 °C steadily increases from 0.18 W∙m^−1^∙K^−1^ for the sample with 5 vol.% PZT filler to 0.36 W∙m^−1^∙K^−1^ for the specimen containing 50 vol.% PZT (Figure 7). Furthermore, the thermal conductivity of the PZT-polymer composites could be satisfactorily modelled by Landauer’s theory of effective percolation with λ_matrix_ = 0.14 W∙m^−1^∙K^−1^ and λ_filler_ = 0.81 W∙m^−1^∙K^−1^. The specimen with 55 vol.% PZT filler should be considered as an outlier due to the difficulties in the homogenization of the starting materials.

### 3.4. Piezoelectric Properties of PZT-Polymer Composites

**Figure 8:**
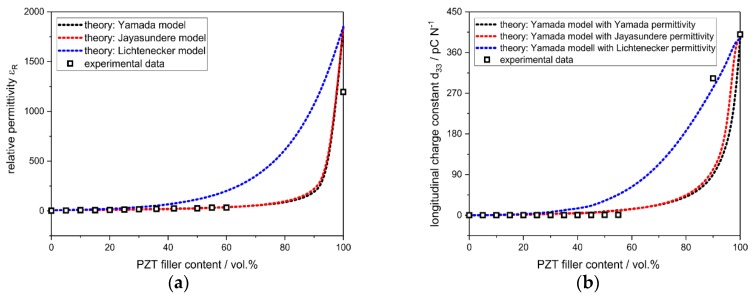
Experimental results for the relative permittivity (**a**) and the piezoelectric charge constant d_33_ (**b**) of PZT-polymer composites, the pure polymer matrix, and the pure PZT filler. The experimental data could be modelled sufficiently using the models of Yamada and Jayasundere for the extrapolation of the permittivity and d_33_ data.

The relative permittivity ε_R_ of the samples increases exponentially with an increasing amount of PZT material (Figure 8a). For the pure polymer matrix, a low ε_R_ of 2.34 is the result, which increases to 1195 for the pure PZT filler. The course of ε_R_, with the increasing volume fraction of PZT filler, is predicted satisfactorily by both the Yamada model for oriented anisotropic particles but without particle–particle interaction [10] and the model of Jayasundere, which assumes spherical particles, but includes interactions between the filler particles [11]. The simple Lichtenecker rule of mixture overestimates the permittivity of the composite for filler contents above 30 vol.%. The longitudinal charge constant d_33_ increases almost linearly from 0.03 pC∙N^−1^ for the PZT-polymer composite with 5 vol.% PZT filler to a value 0.77 pC∙N^−1^ for the sample with 55 vol.% PZT content (Figure 8b), and the pure PZT filler gives a d_33_ of 400 pC∙N^−1^, which is in good agreement with the specifications of the manufacturer (443 pC∙N^−1^; Noliac Ceramics s.r.o.). The course of the d_33_ constant as a function of the PZT load is sufficiently modelled with the models of Yamada and Jayasundere as well. Nevertheless, the results for the longitudinal charge constant for the PZT-polymer composites are significantly lower compared to data for PZT-PDMS composites with similar filler loads. For these, a maximal d_33_ of 25 pC∙N^−1^ at a filler load of 50 vol.% has been observed [13]. Most likely, this is a consequence of the different sample geometry (thin films rather than bulk specimens) and the polarization, which was performed at a higher field strength of 12 kV∙mm^−1^ compared to 2 kV∙mm^−1^ within the present work. 

## 4. Conclusions

The main goal of the present work was to show the feasibility of a preceramic polymer as a suitable matrix phase for PZT-polymer composites. It could be shown that the manufacturing of composites with lead zirconate titanate (PZT) as functional filler phase and a silsesquioxane polymer as thermally curable matrix phase is possible. A filler load of 55 vol.%, which is approximately 90 wt.%, could be achieved. The mechanical strength of the PZT-polymer composite materials ranging between 15 and 29 MPa was slightly lower than that of the pure matrix and the filler materials and correlated with the degree of homogenization of the starting components. The composite’s thermal conductivity is a direct function of the filler load according to Landauer’s theory of effective percolation, lying in between the values for the pure matrix material and the PZT filler, respectively. The thermal stability of the PZT-PDC composites has been proven up to a temperature of 750 °C; higher temperatures result in the decomposition of the PZT filler under the volatization of PbO and formation of ZrO_2_. From XRD studies, an inhibition of the crystallization of the PDC matrix in the presence of the PZT filler has been observed; conversely, the embedment of PZT into the PDC matrix results in a pronounced decomposition of the filler compared to the pure PZT material. This effect has been observed for other silicon-based PDCs with inorganic fillers as well as, for example, in composites of SiOC as a matrix phase and LiAlSiO_4_ (β-eucryptite) or ZrW_2_O_8_ (zirconium wolframate) as fillers [29,30]. As an analogy to this study, the formation of SiO_2_ from the PDC matrix has been accounted for the influence on the phase stability of the respective inorganic filler phase. The piezoelectric properties of the composite materials are very limited with low values of the piezoelectric charge and voltage parameters d_33_ and g_33_, respectively. Essentially, this is a consequence of the polarization procedure, performed at a lower field strength compared to similar composites.

In sum, this study was intended as a proof of principle for the successful incorporation of a PZT filler into a preceramic polymer or into the respective SiOC PDC matrix generated therefrom. Future studies should be aimed at the investigation of effects related to the degree of crosslinking in the preceramic polymer, which could be modified by the crosslinking temperature and duration, on the one hand side and the role and composition of the amorphous phase formed during the matrix-induced decomposition of the PZT filler. These effects could then be correlated to the properties of this class of composites, resulting in a more comprehensive understanding of the structure–property relations in this material system.

## Figures and Tables

**Table 1:** Phase composition and chemical composition of the individual PZT fillers for selected PZT-polymer and PZT-SiOC composites (r-PZT: rhombohedral Pb(Zr*_x_*Ti_1−*x*_)O_3_; t-PZT: tetragonal Pb(Zr*_x_*Ti_1−*x*_)O_3_). The Zr content *x* and the Ti content (1−*x*) in the Pb(Zr*_x_*Ti_1−*x*_)O_3_ filler is calculated by Vegard’s law from the unit cell volume V and applied in the respective Rietveld analyses.

Sample		Phase Content and Chemical Composition of Pb(Zr*_x_*Ti_1−*x*_)O_3_ (PZT) Filler							
filler content/wt.%	pyrolysis T/°C	r-PZT/wt.%	V_r-PZT_/Å^3^	Zr content *x*	t-PZT/wt.%	V_t-PZT_/Å^3^	Zr content *x*	m-ZrO_2_/wt.%	cristobalite/wt.%
50	200	29 ± 1.4	408.5 ± 0.2	0.534 ± 0.004	71 ± 1.4	67.60 ± 0.03	0.522 ± 0.003	-/-	-/-
50	750	34 ± 2.4	408.2 ± 0.3	0.524 ± 0.007	66 ± 2.4	67.53 ± 0.04	0.515 ± 0.005	-/-	-/-
50	1250	-/-	-/-	-/-	19 ± 0.3	63.70 ± 0.01	0.096 ± 0.001	81 ± 0.3	-/-
50	200	33 ± 1.0	408.2 ± 0.2	0.528 ± 0.004	67 ± 1.0	67.58 ± 0.02	0.520 ± 0.002	-/-	-/-
100	200	38.7 ± 0.5	407.0 ± 0.6	0.502 ± 0.013	61.3 ± 0.5	67.40 ± 0.01	0.501 ± 0.001	-/-	-/-
0	1250	-/-	-/-	-/-	-/-	-/-	-/-	-/-	100 ^a^
50	1250	-/-	-/-	-/-	18.1 ± 0.2	63.69 ± 0.01	0.095 ± 0.001	81.9 ± 0.2	-/-
100	1250	1.3 ± 0.2	385.0 ± 0.5	0.02 ± 0.01	59.5 ± 0.5	64.92 ± 0.01	0.229 ± 0.001	39.2 ± 0.5	-/-

^a^ of the crystalline fraction; a broad maximum below 18° indicates a significant amount of amorphous material.

## Supplementary Materials

The following are available online at https://www.mdpi.com/1996-1944/13/7/1520/s1, Figure S1: SEM micrographs of PZT-polymer composites with increasing filler content (dark: polymer matrix, bright: filler particles); (a) 5 vol.% PZT, (b) 15 vol.% PZT, (c) 25 vol.% PZT, (d) 35 vol.% PZT, (e) 45 vol.% PZT, (f) 55 vol.% PZT, Figure S2: SEM micrographs of selected PZT-polymer composites with a filler content of 40 vol.% PZT with increasing heat treatment processing; (a) 500 °C, (b) 750 °C, (c) 1000 °C, (d) 1250 °C, Figure S3: Particle size distribution of the PZT powder used within this study.

## References

[1] Babu I., van den Ende D.A., de With G. (2010). Processing and characterization of piezoelectric 0-3 PZT/LCT/PA composites. J. Phys. D Appl. Phys..

[2] Salmang H., Scholze H., Telle R. (2007). Keramik.

[3] Jaffe B., Cook W.R., Jaffe H. (1971). Piezoelectric Ceramics.

[4] Seifert K., Schlegel T., Rödel J. (2006). Entwicklung neuer oxidischer Piezowerkstoffe. Thema Forschung.

[5] Newnham R.E., Skinner D.P., Cross L.E. (1978). Connectivity and piezoelectric-pyroelectric composites. Mater. Res. Bull..

[6] Konegger T., Potzmann R., Puchberger M., Liersch A. (2011). Matrix–filler interactions in polysilazane-derived ceramics with Al_2_O_3_ and ZrO_2_ fillers. J. Eur. Ceram. Soc..

[7] Sharma S.K., Gaur H., Kulkarni M., Patil G., Bhattacharya B., Sharma A. (2013). PZT–PDMS composite for active damping of vibrations. Compos. Sci. Technol..

[8] Babu I., Hendrix M.M.R.M., de With G. (2014). PZT-5A4/PA and PZT-5A4/PDMS piezoelectric composite bimorphs. Smart Mater. Struct..

[9] Bhimasankaram T., Suryanarayana S., Prasad G. (1998). Piezoelectric polymer composite materials. Curr. Sci..

[10] Yamada T., Ueda T., Kitayama T. (1982). Piezoelectricity of a high-content lead zirconate titanate/polymer composite. J. Appl. Phys..

[11] Jayasundere N., Smith B.V. (1993). Dielectric constant for binary piezoelectric 0-3 composites. J. Appl. Phys..

[12] Satish B., Sridevi K., Vijaya M.S. (2002). Study of piezoelectric and dielectric properties of ferroelectric PZT-polymer composites prepared by hot-press technique. J. Phys. D Appl. Phys..

[13] Babu I., de With G. (2014). Highly flexible piezoelectric 0–3 PZT–PDMS composites with high filler content. Compos. Sci. Technol..

[14] Greil P. (2000). Polymer Derived Engineering Ceramics. Adv. Eng. Mater..

[15] Furukawa T., Fujino K., Fukada E. (1976). Electromechanical Properties in the Composites of Epoxy Resin and PZT Ceramics. Jpn. J. Appl. Phys..

[16] Landauer R. (1952). The Electrical Resistance of Binary Metallic Mixtures. J. Appl. Phys..

[17] Smith D.S., Alzina A., Bourret J., Nait-Ali B., Pennec F., Tessier-Doyen N., Otsu K., Matsubara H., Elser P., Gonzenbach U.T. (2013). Thermal conductivity of porous materials. J. Mater. Res..

[18] Song B.-M., Kim D.-Y., Shirasaki S.-I., Yamamura H. (1989). Effect of Excess PbO on the Densification of PLZT Ceramics. J. Am. Ceram. Soc..

[19] Vegard L. (1921). Die Konstitution der Mischkristalle und die Raumfllung der Atome. Z. Physik.

[20] Frantti J., Lappalainen J., Eriksson S., Lantto V., Nishio S., Kakihana M., Ivanov S., Rundlöf H. (2000). Neutron Diffraction Studies of Pb(Zr_x_Ti_1−x_)O_3_ Ceramics. Jpn. J. Appl. Phys..

[21] Joseph J., Vimala T.M., Sivasubramanian V., Murthy V.R.K. (2000). Structural investigations on Pb(Zr_x_Ti_1−x_)O_3_ solid solutions using the X-ray Rietveld method. J. Mater. Sci..

[22] Mastelaro V.R., Doriguetto A.C., Neves P.P., Garcia D., Lente M.H., Mascarenhas Y.P., Michalowicz A., Eiras J.A. (2011). Structural Characterization of Pb_1−x_Ba_x_Zr_0.65_Ti_0.35_O_3_ Ferroelectric Ceramics. Ferroelectrics.

[23] Glazer A.M., Mabud S.A. (1978). Powder profile refinement of lead zirconate titanate at several temperatures. II. Pure PbTiO_3_. Acta Crystallogr. Sect. B Struct. Crystallogr. Cryst. Chem..

[24] Yokota H., Zhang N., Taylor A.E., Thomas P.A., Glazer A.M. (2009). Crystal structure of the rhombohedral phase of PbZr_1−x_Ti_x_O_3_ ceramics at room temperature. Phys. Rev. B.

[25] Fett T., Munz D., Thun G. (1999). Tensile and bending strength of piezoelectric ceramics. J. Mater. Sci. Lett..

[26] Fett T., Munz D., Thun G. (2003). Bending strength of a PZT ceramic under electric fields. J. Eur. Ceram. Soc..

[27] Malič B., Kosec M., Kosmač T. (1992). Mechanical and electric properties of PZT-ZrO_2_ composites. Ferroelectrics.

[28] Kallaev S.N., Gadzhiev G.G., Kamilov I.K., Omarov Z.M., Sadykov S.A., Reznichenko L.A. (2006). Thermal properties of PZT-based ferroelectric ceramics. Phys. Solid State.

[29] Fedorova A., Betke U., Scheffler M. (2017). Polymer Derived Ceramics with b-Eucryptite Fillers: Filler-Matrix Interactions. Adv. Eng. Mater..

[30] Fedorova A., Scheffler M. (2019). Polymer Derived Ceramics with Negative Thermal Expansion Fillers: Zirconium Tungstate. Adv. Eng. Mater..

