# Supplementary material for: Phase Evolution, Filler-Matrix Interactions, and Piezoelectric Properties in Lead Zirconate Titanate (PZT)-Filled Polymer-Derived Ceramics (PDCs)
Source: Materials (Basel). 2020 Mar 26;13(7):1520. doi: 10.3390/ma13071520 (PMC7177491; doi:10.3390/ma13071520)
Supplement: Supplementary file 1 [file materials-13-01520-s001.pdf]

materials

Supporting Information

Phase Evolution, Filler-Matrix Interactions, and Piezoelectric Properties in Lead Zirconate Titanate (PZT)-Filled Polymer-Derived Ceramics (PDCs)

Franziska Eichhorn [1], Simone Kellermann [1], Ulf Betke [2], Tobias Fey [1,3,*]

[1] Institute of Glass and Ceramics, Department of Materials Science and Engineering, University of Erlangen-Nürnberg, Martensstraße 5, 91058 Erlangen, Germany; franziska.eichhorn@fau.de (F.E.); kellermann_simone@web.de (S.K.)

[2] Institute for Materials and Joining Technology—Nonmetallic Inorganic Materials and Composites, Otto-von-Guericke-University Magdeburg, Große Steinernetischstraße 6, 39104 Magdeburg, Germany; ulf.betke@ovgu.de

[3] Frontier Research Institute for Materials Science, Nagoya Institute of Technology, Nagoya 466-8555, Japan

* Correspondence: tobias.fey@fau.de

Received: 26 February 2020; Accepted: 23 March 2020; Published: date

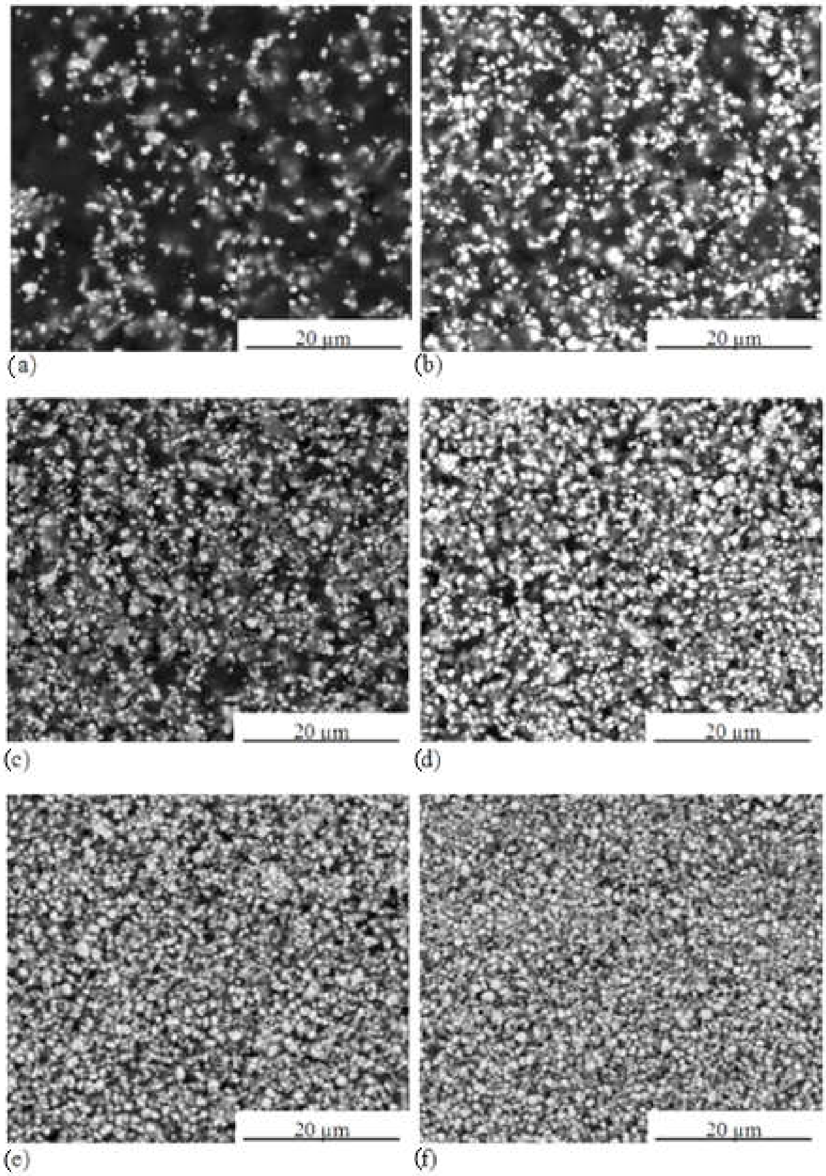

Figure S1. SEM micrographs of PZT-polymer composites with increasing filler content (dark: polymer matrix, bright: filler particles); (**a**) 5 vol.% PZT, (**b**) 15 vol.% PZT, (**c**) 25 vol.% PZT, (**d**) 35 vol.% PZT, (**e**) 45 vol.% PZT, (**f**) 55 vol.% PZT.

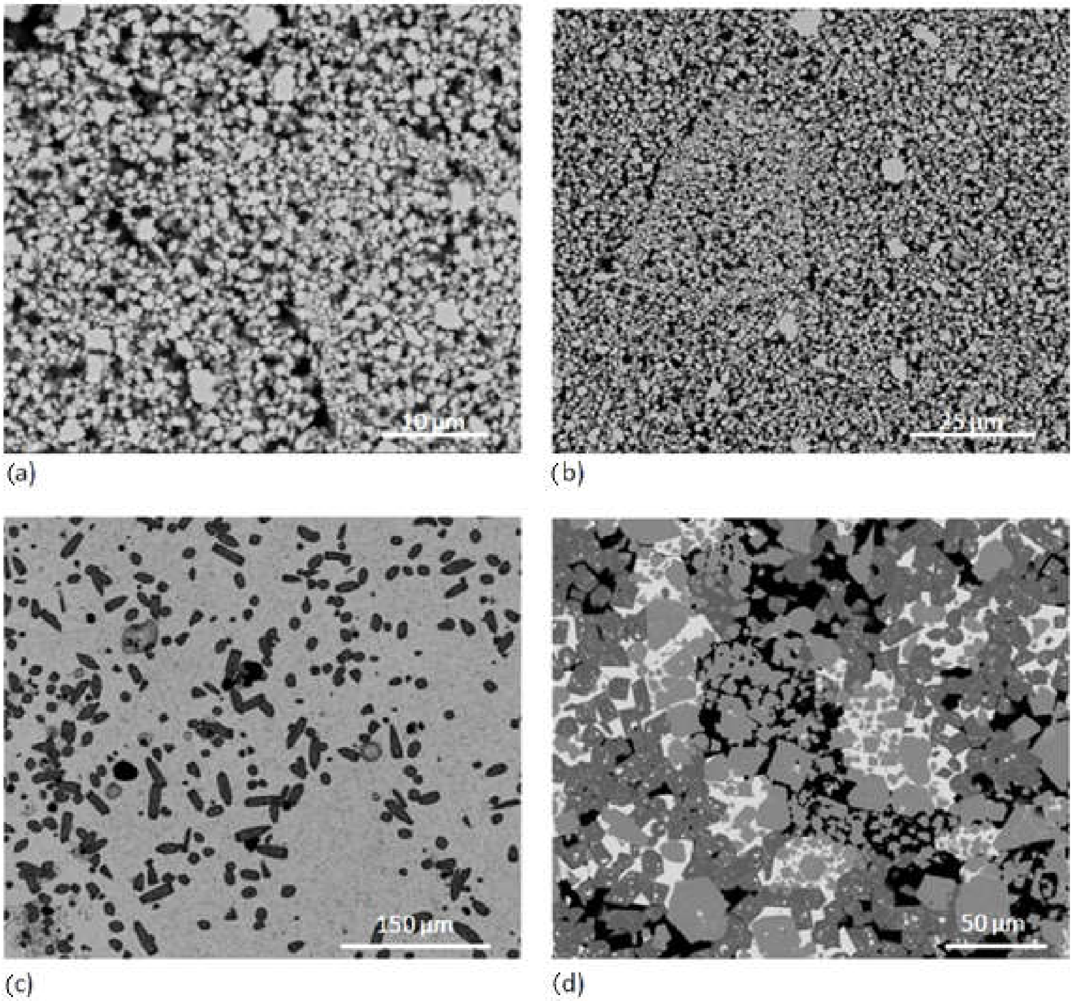

Figure S2. SEM micrographs of selected PZT-polymer composites with with a filler content of 40 vol.% PZT with increasing heat treatment processing; (**a**) 500 °C, (**b**) 750 °C, (**c**) 1000 °C, (**d**) 1250 °C.

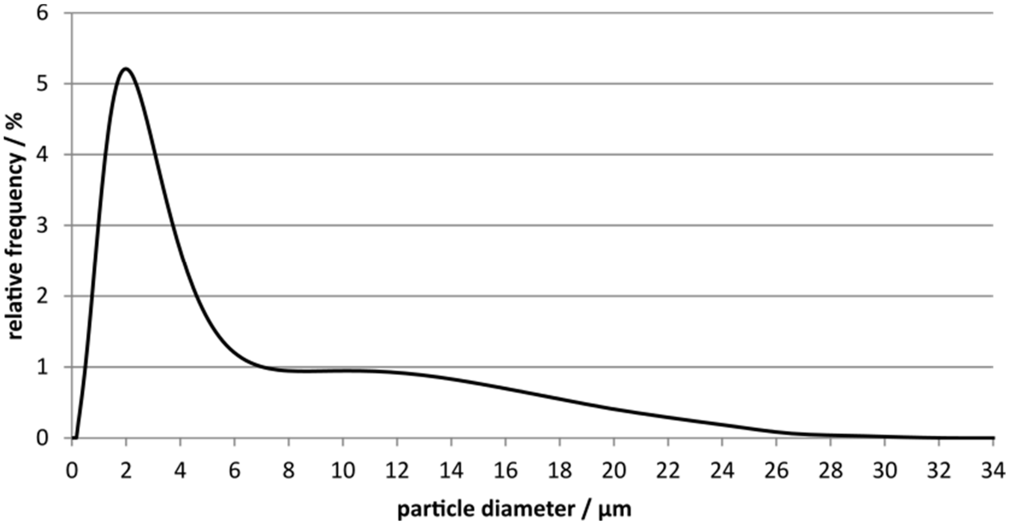

Figure S3. Particle size distribution of the NCE51 PZT-powder

© 2020 by the authors. Submitted for possible open access publication under the erms and conditions of the Creative Commons Attribution (CC BY) license http://creativecommons.org/licenses/by/4.0/).
